# Age-Related Outcomes in Heart Failure with Mildly Reduced Ejection Fraction

**DOI:** 10.3390/jcm13175151

**Published:** 2024-08-30

**Authors:** Marielen Reinhardt, Tobias Schupp, Michael Behnes, Felix Lau, Alexander Schmitt, Noah Abel, Muharrem Akin, Jonas Rusnak, Ibrahim Akin, Kathrin Weidner

**Affiliations:** 1 Department of Cardiology, Angiology, Haemostaseology and Medical Intensive Care, University Medical Centre Mannheim, Medical Faculty Mannheim, Heidelberg University, 68167 Mannheim, Germany; marielen.reinhardt@stud.uni-heidelberg.de (M.R.); michael.behnes@umm.de (M.B.); felix.lau@stud.uni-heidelberg.de (F.L.); alexander.schmitt02@stud.uni-heidelberg.de (A.S.); noah.abel@stud.uni-heidelberg.de (N.A.); ibrahim.akin@umm.de (I.A.); kathrin.weidner@umm.de (K.W.); 2 Department of Cardiology, St. Josef-Hospital, Ruhr-Universität Bochum, 44791 Bochum, Germany; akin.muharrem@klinikum-bochum.de; 3 Department of Cardiology, Angiology and Pneumology, University Hospital Heidelberg, 69120 Heidelberg, Germany; jonas.rusnak@med.uni-heidelberg.de

**Keywords:** heart failure, ejection fraction, HFmrEF, age, elderly, mortality

## Abstract

**Objective:** This study investigates age-related differences and outcomes in patients hospitalized with heart failure with a mildly reduced ejection fraction (HFmrEF). **Background:** The characterization of patients with HFmrEF and the prognostic value of age has rarely been investigated. **Methods:** Patients with HFmrEF were retrospectively included at one institution between 2016 and 2022. The distribution of HF aetiology and prognostic outcomes were investigated comparing patients with ≤40, >40 to ≤60, >60 to ≤80, and >80 years of age. The primary endpoint was long-term all-cause mortality. Kaplan–Meier and multivariable Cox proportional regression analyses were applied for statistics. **Results:** For the present study, 2184 patients with HFmrEF with a median age of 76 years were included. Non-ischemic cardiomyopathy was the most common HF aetiology in patients <40 years of age, whereas patients with 60–80 years of age (60.2%) and >80 years of age (58.2%) had the higher rates of ischemic cardiomyopathies. The risk of long-term all-cause mortality at 30 months was highest in patients with >80 years of age (HR = 2.167; 95% CI 1.928–2.436; *p* = 0.001), even after multivariable adjustment. Furthermore, patients with >80 years of age had the highest risk of HF-related rehospitalization (HR = 1.529; 95% CI 1.293–1.807; *p* = 0.001). **Conclusions:** Ischemic cardiomyopathy represents the most common cause of HF in elderly patients with HFmrEF, whereas younger patients were more likely to suffer from non-ischemic HF aetiologies. Increasing age was an independent predictor of long-term all-cause mortality in patients hospitalized with HFmrEF.

## 1. Introduction

The prevalence of chronic heart failure (HF) is significantly increasing, related to demographic changes and an overall ageing population [1]. It is estimated that HF affects more than 64 million people worldwide with a corresponding prevalence of up to 4% in the adult population and 5-year mortality rates of 25–80% [2]. HF with mildly reduced ejection fraction (HFmrEF), i.e., left ventricular ejection fraction (LVEF) from 41% to 49%, is an underrecognized subtype of HF [3]. Previous HF registries and randomized controlled trials (RCTs) typically excluded those patients, which has resulted in a gap of data regarding underlying characteristics as well as prognostic outcomes of patients with HFmrEF [4].

Ongoing demographic changes including an overall ageing is associated with an increased incidence and prevalence of HF due to structural and functional changes in the cardiovascular system [5]. Accordingly, morbidity and mortality of patients with HF was shown to increase with age [6]. Demographic changes challenge our health system, as we are confronted with more elderly people. People over 80 years of age represent the most rapidly expanding group in Europe; for instance, the proportion of octogenarians grew from 5.4% to 7.2% in the last decade [7,8]. At the same time, an increased incidence of HF was observed in younger adults as well, which may be related to a higher prevalence of risk factors such as diabetes mellitus and obesity [9]. However, even though studies show a slight decrease in age-related prevalence, the prevalence in the group of octogenarians is up to almost 15% [7,10]. 

The aetiology of HF is different according to patients’ ages and implicates different underlying comorbidities, clinical characteristics, and pathophysiological mechanisms leading to HF [11]. In fact, Lainščak et al. suggested that guideline-directed treatment was lower with ageing of HF patients, suggesting an undertreatment of elderly HF patients [12]. Furthermore, data about the use of device and interventional therapy in elderly HF patients remains controversial, although the implantation of cardiac devices as well as coronary intervention may positively affect outcomes of patients with HF [13,14]. While other studies focused on age-related differences across the spectrum of LVEF, fewer data are available concerning the role of age in HFmrEF patients [5,15]. Therefore, a better characterization of elderly patients and the correct treatment according to age-related differences in the HFmrEF group is deemed necessary. Previous research on HFmrEF has predominantly relied on studies with stringent inclusion criteria and pre-selection bias, most being performed as post-hoc studies from RCTs. While trials with pre-selection criteria, like New York Heart Association (NYHA) stage, amino-terminal prohormone of brain natriuretic peptide (NT-proBNP) levels, and concomitant comorbidities, provide valuable information such as the efficacy of specific medical treatment of HF, they may also potentially limit the generalizability of the findings to the real-world clinical practice [15,16]. This study is based on an all-comers design, aiming to provide a broader approach and inclusion of patients with HFmrEF without pre-selection bias and consequently reflecting the heterogeneity within the HFmrEF cohort.

The present study sought to investigate (1) the distribution of HF aetiology stratified by age, as well as (2) the prognostic value of age with regard to the primary endpoint all-cause mortality using a large sample cohort of patients hospitalized with HFmrEF.

## 2. Methods

### 2.1. Study Patients, Design and Data Collection 

All patients hospitalized with HFmrEF at one University Medical Centre between January 2016 and December 2022 were included retrospectively. Specific data regarding the identification and documentation of patient-related data and outcomes were recently published [17]. The present sub-study was derived from the “Heart Failure With Mildly Reduced Ejection Fraction Registry” (HARMER), which represents a retrospective single-centre registry including consecutive patients with HFmrEF hospitalized at the University Medical Centre Mannheim (UMM), Germany (clinicaltrials.gov identifier: NCT05603390). The registry was carried out according to the principles of the declaration of Helsinki and was approved by the medical ethics committee II of the Medical Faculty Mannheim, University of Heidelberg, Germany (ethical approval code: 2022-818, approval date: 4 April 2022).

### 2.2. Inclusion, Exclusion Criteria, Risk Stratification

For the present sub-study, patients with HFmrEF with at least 18 years of age were included. Standardized transthoracic echocardiography during index hospitalization was performed in all patients. The diagnosis of HFmrEF was determined in accordance with the “2021 European Society of Cardiology (ESC) Guidelines for the diagnosis and treatment of acute and chronic HF” [18] and patients with a LVEF between 41 and 49% with additional symptoms and/or signs of HF were included. For the present study, the presence of right ventricular dysfunction was defined as a tricuspid annular plane systolic excursion (TAPSE) <18 mm. Chronic kidney disease (CKD) was defined as abnormalities of kidney function with implication for health accompanied by an estimated glomerular filtration rate (eGFR) <60 mL/min/1.73 m^2^ (GFR categories G3a-G5) and a duration >3 months [19]. For the present study, the HF aetiology was assessed as recently published [20].

Risk stratification was performed according to patients’ age on admission and patients were stratified by ≤40, >40 to ≤60, >60 to ≤80, and >80 years of age. 

### 2.3. Study Endpoints

The primary endpoint was long-term all-cause mortality. Long-term was defined as the median time of clinical follow-up (i.e., 30 months). Secondary endpoints comprised in-hospital all-cause mortality, all-cause mortality at 12 months. Further secondary endpoints included rehospitalization for worsening HF, cardiac rehospitalization, acute myocardial infarction (AMI), stroke, coronary revascularization, and major adverse cardiac and cerebrovascular events (MACCE) at long-term follow-up. All-cause mortality was documented using the electronic hospital information system and by directly contacting state resident registration offices (‘Bureau of Mortality Statistics’). HF-related hospitalization was defined as a rehospitalization due to worsening HF requiring intravenous diuretic therapy. Cardiac rehospitalization was defined as rehospitalization due to a primary cardiac condition, including worsening HF, AMI, coronary revascularization and symptomatic atrial or ventricular arrhythmias. MACCE was defined as composite of all-cause mortality, coronary revascularization, non-fatal AMI, and non-fatal stroke. All rehospitalizations at our institution including, the rates of rehospitalization for HF, AMI, coronary revascularization, and stroke were documented at our institution.

### 2.4. Statistical Methods

Quantitative data were demonstrated in the median and interquartile range (IQR), and ranged depending on the distribution of the data. Four age-dependent pre-specified subgroups were compared using the Kruskal–Wallis test. In the case of a statistically significant result, Dunn’s tests were performed for pairwise comparisons (i.e., Mann–Whitney U-tests with Bonferroni correction in order to control the type I error). Deviations from a Gaussian distribution were tested using the Kolmogorov–Smirnov test. Qualitative data are presented as absolute and relative frequencies and were compared using the Chi-square test or the Fisher’s exact test, as appropriate. Pairwise comparisons were performed using Bonferroni correction. Kaplan–Meier analyses were performed stratified by age, and univariable hazard ratios (HRs) were given together with 95% confidence intervals (CIs). Using multivariable Cox regression analyses, the prognostic value of age was investigated using pre-selected age-groups (i.e., ≤40, >40 to ≤60, >60 to ≤80, and >80 years of age), as well as an age per decade increase and as a continuous variable (i.e., per year increase). Multivariable Cox regression models were applied using the “forward selection” option. Proportional hazard assumptions for all variables were checked using log-minus-log plots and Schoenfeld residuals. Cox regression analyses were applied within the entire study cohort, as well as in pre-specified subgroups, stratified by sex, the presence or absence of ischemic cardiomyopathy (ICM), NYHA functional class, as well as stratified by right ventricular function reflected by TAPSE. Results of all statistical tests were considered significant for *p* ≤ 0.05. SPSS (Version 28, IBM, Armonk, New York, NY, USA) was used for statistics.

## 3. Results

### 3.1. Study Population

From 2016 to 2022, 2228 consecutive patients with HFmrEF were hospitalized at our institution. A total of 44 patients (1.97%) were lost to follow-up and excluded from the present study. The final study comprised 2184 patients presenting with HFmrEF with a median age of 76 years (mean 72; IQR 72–73 years of age). When stratified by four pre-specified age groups, patients >80 years of age were less commonly males (55.7% vs. 66.6% vs. 77.3% vs. 69.4%; *p* = 0.001) and presented with higher rates of pre-existent coronary artery disease (CAD) (46.9% vs. 43.2% vs. 27.2% vs. 6.5%; *p* = 0.001), prior AMI (25.7% vs. 25.0% vs. 19.3% vs. 6.5%; *p* = 0.001), congestive HF (40.2% vs. 32.7% vs. 26.6% vs. 17.7%; *p* = 0.001), and CKD (46.2% vs. 27.6% vs. 12.1% vs. 8.1%; *p* = 0.001) (Table 1). Furthermore, the patients >80 years of age had higher rates of prior percutaneous coronary intervention (PCI) (30.2% vs. 30.1% vs. 20.5% vs. 6.5%; *p* = 0.001) and coronary artery bypass graft (11.9% vs. 10.6% vs. 4.2% vs. 0.0%; *p* = 0.001). Regarding the distribution of cardiovascular risk factors, patients >80 years had higher rates of arterial hypertension (86.9% vs. 21.0%; *p* = 0.001) whereas patients aged >60 to ≤80 presented with higher rates of diabetes mellitus (39.8% vs. 9.7%; *p* = 0.001) and hyperlipidaemia (33.0% vs. 4.8%; *p* = 0.001) as compared to patients ≤40 years of age. On the contrary, the rates of active smoking were highest in patients aged >40 to ≤60 years (42.6%) and lowest in patients aged >80 years (4.0%) (*p* = 0.001). 

Regarding comorbidities during index hospitalization, patients aged >40 to ≤60 years had the highest rates of unstable angina (5.7%; *p* = 0.018) and ST-segment elevation AMI (STEMI) (17.8%; *p* = 0.001), whereas rates of acute decompensated HF (32.2%; *p* = 0.001) and atrial fibrillation (56.5%; *p* = 0.001) were highest in patients >80 years, as can be seen in Table 1.

As outlined in Table 2, patients >80 years of age had higher interventricular septal end diastole (12 vs. 10 mm; *p* = 0.001) and left atrial diameter (45 vs. 36 mm; *p* = 0.001) but lower left ventricular end-diastolic diameter (48 vs. 50 mm; *p* = 0.001) and TAPSE (19 vs. 21 mm; *p* = 0.001), as compared to patients aged ≤40. Furthermore, the rates of diastolic dysfunction (76.8% vs. 74.0% vs. 63.4% vs. 29.0%; *p* = 0.001) and valvular heart diseases, such as moderate–severe aortic stenosis (AS) (18.8% vs. 6.8% vs. 0.0% vs. 1.6%; *p* = 0.001), moderate–severe mitral regurgitation (MR) (19.2% vs. 9.9% vs. 3.9% vs. 1.6%; *p* = 0.001), and moderate–severe tricuspid regurgitation (TR) (27.6% vs. 12.2 s. 2.7% vs. 1.6%; *p* = 0.001) were higher in patients >80 years of age compared to younger patients. 

A higher proportion of patients aged >40 to ≤60 underwent invasive coronary angiography during hospitalization compared to patients >80 years of age (55.9% vs. 29.2%; *p* = 0.001). However, when comparing the two groups, patients >80 years of age had higher rates of 3-vessel CAD (45.5% vs. 33.5%; *p* = 0.001). Finally, angiotensin-converting enzyme inhibitors (ACEi) were more frequently prescribed to patients aged >40 to ≤60 compared to the oldest patient group (57.4% vs. 46.0%; *p* = 0.005), whereas angiotensin receptor blocker (ARB) rates were higher in patients >80 years of age (27.8% vs. 15.2%; *p* = 0.001). Sodium glucose linked transporter 2 (SGLT2) inhibitors were more commonly prescribed to patients aged >40 to ≤60 (5.8% vs. 2.0%; *p* = 0.003).

### 3.2. Distribution of HF Aetiologies Stratified by Age

**Figure 1:**
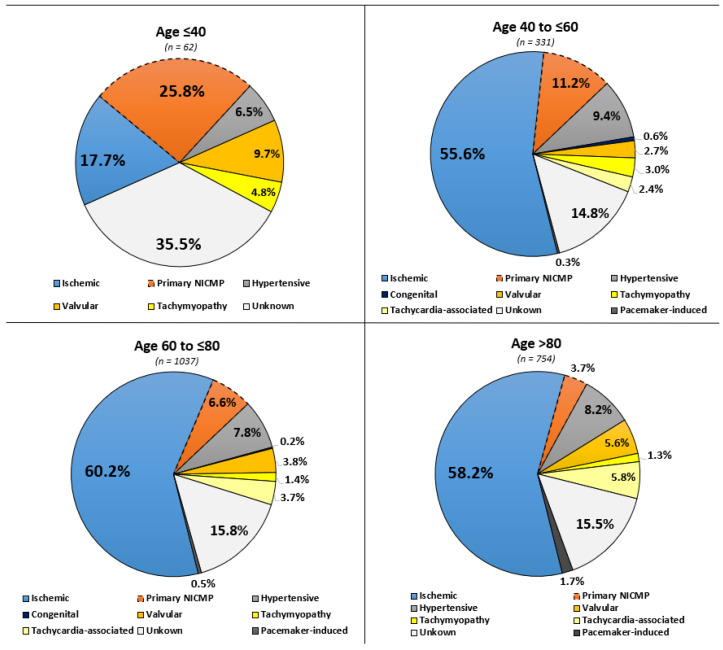
Distribution of heart failure aetiologies stratified by patients’ age.

The most common aetiology in patients >40 years of age was ICM, with a prevalence of 55.6% of patients >40 to ≤60 years, 60.2% of patients >60 to ≤80 years, and 58.2% of patients with >80 years of age (Figure 1). In contrast, in patients ≤40 years, 35.5% had HF with an unknown cause followed by 25.8% of patients with primary non ischemic cardiomyopathy (NICM). Accordingly, rates of NICM were higher in younger compared to elderly patients (25.8% vs. 11.2% vs. 6.6% vs. 3.7%; *p* = 0.001).

### 3.3. Prognostic Impact of Age in Patients with HFmrEF

**Figure 2:**
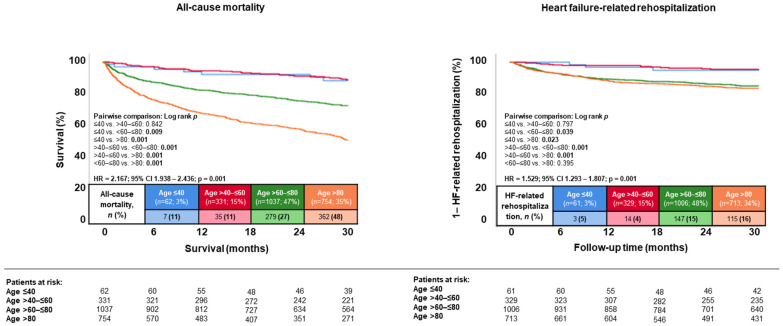
Kaplan–Meier analyses comparing the prognostic impact of age on the risk of all-cause mortality (**left panel**) and hospitalization for worsening HF (**right panel**) in patients with HFmrEF.

**Figure 3:**
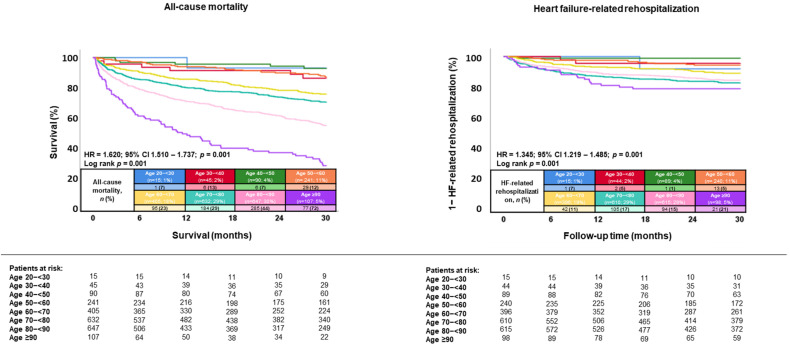
Kaplan–Meier analyses comparing the prognostic impact of age stratified by decades on the risk of all-cause mortality (**left panel**) and hospitalization for worsening HF (**right panel**) in patients with HFmrEF.

During a median follow-up of 30 months (IQR 12–54 months), the primary endpoint all-cause mortality occurred in 48.0% of patients >80 years, in 26.9% of patients >60 to ≤80, in 10.6% of patients >40 to ≤60 years, and in 11.3% of patients aged ≤40 years (*p* = 0.001) (Figure 2; left panel). Accordingly, patients >80 years of age had the highest risk of all-cause mortality at 30 months compared to younger patients (i.e., patients >60–≤80, >40–≤60, and ≤40) (HR = 2.167; 95% CI 1.928–2.436; *p* = 0.001). Even when stratified by decades of age, a higher age was associated with increased risk of all-cause mortality (HR = 1.620; 95% CI 1.510–1.737; *p* = 0.001; log rank *p* = 0.001) as well as HF-related rehospitalization (HR = 1.345; 95% CI 1.219–1.485; *p* = 0.001; log rank *p* = 0.001) in patients hospitalized with HFmrEF (Figure 3).

Furthermore, the risk of HF-related rehospitalization at 30 months was higher in patients >80 years of age (16.1% vs. 14.6% vs. 4.3% vs. 4.9%; *p* = 0.001; HR = 1.529; 95% CI 1.293–1.807; *p* = 0.001) (Figure 2; right panel). With regard to the secondary key endpoints, a higher age was associated with a higher risk of in-hospital mortality (5.4% vs. 3.0% vs. 0.6% vs. 1.6%; *p* = 0.031; HR = 1.603; 95% CI 1.109–2.316; *p* = 0.012) and MACCE at 30 months (51.6% vs. 35.6% vs. 22.1% vs. 16.1%, *p* = 0.001; HR = 1.667; 95% CI 1.511–1.838; *p* = 0.001) (Table 3).

After multivariable adjustment, patients >80 years of age (HR = 3.874; 95% CI 1.770–8.478; *p* = 0.001) and patients with >60 to ≤80 years of age (HR = 2.211; 95% CI 1.020–4.790; *p* = 0.044) were still associated with a higher risk of 30-month all-cause mortality compared to patients ≤40 years (reference group) (Table 4). Besides age, a higher body mass index (BMI) (HR = 0.952; 95% CI 0.934–0.970; *p* = 0.001), hyperlipidaemia (HR = 0.705; 95% CI 0.577–0.861; *p* = 0.001), and ICM (HR = 0.770; 95% CI 0.646–0.918; *p* = 0.004) were associated with a lower risk for 30-month all-cause mortality. Contrarily, prior chronic HF (HR = 1.223; 95% CI 1.021–1.466; *p* = 0.029), diabetes mellitus (HR = 1.254; 95% CI 1.046–1.505; *p* = 0.015), malignancies (HR = 3.099; 95% CI 2.564–3.746; *p* = 0.001), higher creatinine levels (HR = 1.156; 95% CI 1.091–1.226; *p* = 0.001), acute decompensated HF (HR = 1.611; 95% CI 1.335–1.942; *p* = 0.001), cardiogenic shock (HR = 2.556; 95% CI 1.598–4.087; *p* = 0.001), TAPSE <18 mm (HR = 1.262; 95% CI 1.047–1.521; *p* = 0.014), AS (HR = 1.416; 95% CI 1.116–1.796; *p* = 0.004), and TR (HR = 1.431; 95% CI 1.154–1773; *p* = 0.001) increased the risk of all-cause mortality at 30 months within the entire study cohort. However, the risk of rehospitalization for worsening HF was not affected by patients’ age after multivariable adjustment. The risk of HF-related rehospitalization at 30 months was higher in patients with prior chronic HF (HR = 2.087; 95% CI 1.594–2.732; *p* = 0.001), higher creatinine levels (HR = 1.111; 95% CI 1.013–1.219; *p* = 0.026), atrial fibrillation (HR = 1.913; 95% CI 1.432–2.557; *p* = 0.001), acute decompensated HF (HR = 2.023; 95% CI 1.536–2.664; *p* = 0.001), and moderate or severe AS (HR = 1.682; 95% CI 1.181–2.395; *p* = 0.004). 

When stratified by pre-selected subgroups, a higher age was associated with higher risk of 30-month all-cause mortality in patients with NYHA functional class ≤2 (HR = 1.332; 95% CI 1.055–1.680; *p* = 0.016) and TAPSE ≥18 mm (HR = 1.271; 95% CI 1.010–1.600; *p* = 0.041), whereas older patients with ICM had a higher risk of HF-related rehospitalization (HR = 1.430; 95% CI 1.022–2.001; p = 0.037) (Table 5). 

### 3.4. Changes of LVEF and NT-Pro BNP Levels during Follow-Up

**Figure 4:**
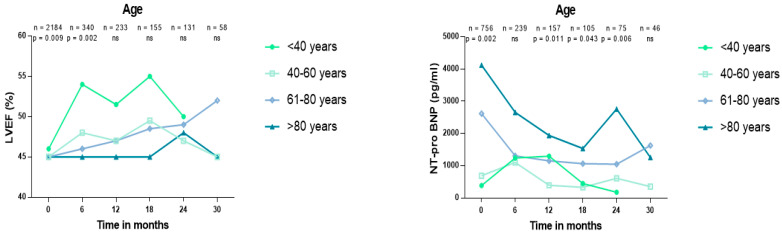
Changes in LVEF (**left panel**) and NT-proBNP levels (**right panel**) among patients stratified by age groups, during 30 months of follow-up. Data are presented as median and interquartile range (IQR).

In patients hospitalized with HFmrEF, LVEF was higher in patients ≤40 years of age compared to patients >40–≤60, >60–≤80, >80 years of age during 6 months of follow-up (54% vs. 48% vs 46% vs. 45%, *p* = 0.002) (Figure 4; left panel). However, LVEF did not significantly differ over 12 (52% vs. 47% vs. 47% vs. 45%), 18 (55% vs. 50% vs. 49% vs. 45%), 24 (50% vs. 47% vs. 49% vs. 48%), and 30 (45% vs. 52% vs. 45% vs. 45%) months of follow-up (*p* >0.005). Furthermore, NT-proBNP levels were higher in patients aged >80 years compared to patients aged >60–≤80, >40–≤60, ≤40 years at 12 (1939 vs. 1154 vs. 395 vs. 1297 pg/mL; *p* = 0.011), 18 (1534 vs. 1064 vs. 333 vs. 449 pg/mL; *p* = 0.043), and 24 (2762 vs. 1048 vs. 610 vs. 177; *p* = 0.006) months of follow-up (Figure 4; right panel).

## 4. Discussion

In patients hospitalized with HFmrEF, the median age was 76 years, which is in line with the findings of Shiga et. al, where HFmrEF patients were shown to be younger than HFpEF patients [21]. On the other hand, the Get With The Guidelines—Heart Failure (GWTG-HF) registry suggested a slightly higher age in patients with HFmrEF [22]. Despite the disparities in age distribution in different registries, higher age is clearly associated with a higher rate of comorbidities that contribute to the development of chronic HF resulting in a multifactorial disease [23]. In fact, previous studies showed that both cardiac and non-cardiac comorbidities increase with higher age regardless of the ejection fraction category [5]. ICM remains one of the most common causes of HFmrEF [15]. Notably, the aetiology of HF in about one third of patients aged ≤40 years remained unknown, which may reflect a combination of the recent introduction of HFmrEF as a previously under-recognized subgroup of HF, with subsequent rather low rates of patients undergoing cardiac magnet resonance imaging, and the inherent challenges in identifying a clear aetiology in a heterogeneous patient population, especially in younger patients. Additionally, in younger patients, especially those with mild symptoms or less severe disease, there may be a focus on managing symptoms rather than thoroughly investigating the underlying cause, which may be supported by the rather high rates of patients with mild HF symptoms in the present study.

However, younger HFmrEF patients show significantly lower rates of ICM compared to older age categories [24]. Subsequently, HF in the young is more commonly attributed to specific factors directly affecting the heart, such as different types of cardiomyopathies and congenital heart disease [25]. This was confirmed in our study, whereas although no evidence of congenital heart disease was found in patients aged ≤40, younger patients presented the highest rates of NICM. In contrast, HF in older patients is more frequently a result of risk factors leading to CAD or AMI and consequently to ICM [25]. In line with this, older patients in our study presented with higher rates of diabetes mellitus, hyperlipidaemia, and peripheral artery disease. Furthermore, elderly patients present with higher rates of concomitant comorbidities, such as CKD, atrial fibrillation, cerebrovascular disease, and anaemia [6]. Accordingly, the highest rates of atrial fibrillation and stroke during index hospitalization were registered among patients >80 years of age, and levels of creatinine and GFR as well as haemoglobin indicated higher incidence of CKD and anaemia in the oldest. Furthermore, in our study cohort, diastolic dysfunction was observed with greater frequency in patients with hypertensive cardiomyopathy compared to those with heart failure of other aetiologies. Additionally, the incidence of hypertensive cardiomyopathy was significantly higher in older patients relative to younger individuals, a finding consistent with the well-established association between arterial hypertension and advanced age. These results likely account for the reduced rates of diastolic dysfunction among younger patients, who are less commonly affected by arterial hypertension. Importantly, while diastolic dysfunction is generally a sign of deteriorating cardiac function with potential prognostic significance, data from the HARMER registry indicated no significant difference in mortality rates between HFmrEF patients with and without diastolic dysfunction [26]. Another relevant comorbidity concerns valvular heart disease, a degenerative process more commonly found in the elderly and related to increased HF risk [25]. MR is considered the most common valvular heart disease in patients >65 years of age and elderly patients account for about 40% of all patients with MR [27]. In our study, moderate–severe MR was the second most common valve degeneration and occurred in 19.2% of patients aged >80 years. The most common valvular disease in our patient cohort was moderate–severe TR, which was found in 27.6% of the oldest patients. Furthermore, moderate–severe AS was highest in patients >80 years of age with a proportion of 18.8%, in line with other studies where AS has been reported in 12 to 26% of patients >75 years of age depending on the measuring criteria employed [27]. 

As reported in the Prospective Multicentre Observational Study of Patients With Heart Failure With Preserved Ejection Fraction (PURSUIT-HFpEF), elderly patients tend to be rehospitalized for worsening HF during follow-up [28]. Furthermore, higher age is associated to an increased risk for worse outcomes and higher mortality [6]. We confirmed these findings for patients >70 to <90 years of age hospitalized with HFmrEF, who had the highest risk of 30-month all-cause mortality and HF-related rehospitalization at 30 months. However, patients aged ≥90 years presented with a significantly lower risk of readmission due to HF worsening as well as all-cause mortality. Notably, the rates of CAD and diabetes mellitus seem to be less common in the very elderly, which may explain our finding suggesting a longer survival of those not suffering from relevant comorbidities associated to HF worsening [6]. Notably, worsening HF itself is a dynamic condition requiring urgent hospitalization and the need of intravenous diuretics, leading to clinical deterioration and a worsening prognosis. The current definition does not include aspects that potentially suggest disease progress, such as the need of higher oral diuretics doses or subclinical worsening, which are commonly recorded in general practice [29]. Patients with worsening HF are more likely to experience adverse clinical events leading to hospitalization, despite optimized medical therapy. Studies showed a reduction in hospitalization for worsening HF in patients treated with further medications, such as repetitive Levosimendan infusions, Vericiguat, Omecamtiv Mecarbil, and Sotagliflozin [29]. Therefore, a focused management of patients with HFmrEF and HF worsening may be beneficial in reducing hospitalization and death, especially in older patients. 

A combination of various disease-modifying factors may contribute to a worse outcome for elderly patients. Reportedly, among prognostic predictors, lower BMI was shown to be associated with worse prognosis in elderly patients, as we could confirm in our study [5,30]. This finding aligns with the ‘obesity paradox’, which suggests that patients with a higher BMI tend to have a better prognosis compared to those with a lower BMI. This paradox has also been observed in patients with HFmrEF [31]. The improved prognosis in obese patients may be linked to molecular and neurohormonal mechanisms that enable them to better manage the catabolic state of heart failure [32]. Additionally, patients with lower BMI are often older and burdened with more cardiovascular comorbidities, contributing to a poorer prognosis [31]. We also found that hyperlipidaemia was associated with a lower risk of mortality. While this may seem counterintuitive, previous studies have shown a positive correlation between higher cholesterol levels and improved survival in heart failure [32]. This could be explained by the endotoxin–lipoprotein hypothesis, which suggests that cholesterol- and triglyceride-rich lipoproteins bind to and neutralize bacterial lipopolysaccharides, potent triggers of inflammatory cytokine release in chronic heart failure [33]. In addition, statin treatment in patients with hyperlipidaemia may provide further benefits due to its immunomodulatory effects [33]. Furthermore, NT-proBNP levels have been defined as useful predictors for HF readmission in octogenarians, although other studies have questioned the value of BNP in older patients, especially as a diagnostic tool [28,34]. Although the outcomes for elderly patients were not adjusted according to NT-proBNP levels, we still found significantly higher levels in patients >80 years of age, which could be related to the rates of acute decompensated HF during index hospitalization in the same patient group.

Evidence-based medical treatment was shown to decrease with ageing, resulting in an under-use of recommended HF drug therapies in the elderly, which may adversely affect outcomes [12]. However, the benefits of HF medication appear to be similar in young patients as well as in older patients and especially renin–angiotensin system (RAS) inhibitors and beta blockers were shown to be associated with a better outcome even in the elderly [6,35]. In our study, more than 75% of patients aged >80 years were discharged with beta blockers, whereas the rates of RAS prescription at discharge (including ACEi and ARB) in patients >80 years of age were similar to the rates in patients aged >40 to <80 years, although ARBs were more commonly prescribed in older patients and ACEi in younger patients. These findings align with data from the Swedish HF (Swede-HF) registry, where the overall use of beta blockers and RAS was registered in up to 86% and 84% of patients, respectively [36]. Furthermore, in our registry, prescription rates of mineralocorticoid receptor antagonist (MRA) were reported in less than 15% of all patients and were similar across all age groups. Data concerning MRA prescription in patients with HFmrEF is inconsistent and reported rates vary from 24 to 58% [36,37]. Generally, even in the updated guidelines no strong recommendations exist for the use of beta blockers, RAS, and MRA in patients with HFmrEF, and as of this moment there are no specific trials for different medication in patients with HFmrEF [38]. However, the use of beta blockers and RAS seems to be mainly established for this patient group, as confirmed by our study. Patients aged >80 years were less likely to be discharged with angiotensin receptor neprilysin inhibitor (ARNI) and SGLT2 inhibitors. While this may suggest an undertreatment with new evidence-based therapies in the elderly, it must be noted that reasons for not prescribing specific HF medication, such as contraindications or intolerances, were not taken into account. Additionally, rates of SGLT2 inhibitors at discharge in our study cohort were low across all age groups, as in line with data from the Swede-HF registry [39]. Notably, the approval for the prescription of SGLT2i for HFmrEF was only obtained in 2023, following new evidence based on the Dapagliflozin Evaluation to Improve the Lives of Patients with Preserved Ejection Fraction Heart Failure (DELIVER) and the Empagliflozin Outcome Trial in Patients with Chronic Heart Failure with Preserved Ejection Fraction (EMPEROR-Preserved) trials, where SGLT2 inhibitors were shown to reduce the risk of cardiovascular death and hospitalization for worsening heart failure in patients with HFmrEF and HFpEF [40,41]. Accordingly, it is expected that the rates of SGLT2 inhibitors will increase following their upgraded level of evidence in 2023 [38]. In future studies, the influence of age on the efficacy of heart failure medications should be investigated following the new standardized treatment protocols. Moreover, studies found lower implantation rates of implantable cardioverter defibrillators (ICDs) and cardiac resynchronization therapy (CRT) but increased rates of pacemaker implantation in elderly patients [42]. Although ICD and CRT implantation rates in our registry were not significantly different across the age groups, the rates of prior pacemakers were in fact higher in patients >80 years of age. Generally, no strong recommendations exist for the use of guideline-recommended medical treatment and device therapy in HFmrEF as of this moment, and to the best knowledge of the authors, no specific trials for different therapies in patients with HFmrEF are available [18]. From this perspective, further studies are needed to investigate the impact of medical and device therapy of HFmrEF according to patients’ age.

Finally, older patients are less likely to undergo invasive coronary intervention, due to fear of adverse events and perceptions of reduced life expectancy [13]. In line with this, only 29% of patients >80 years of age in our study underwent coronary angiography, although more than 50% were treated with PCI and more than 45% presented with 3-vessel CAD. The small proportion of elderly patients who underwent invasive coronary angiography in the first place reflects the undertreatment of a patient group which may have profited from interventional therapy. In fact, the Functional versus Culprit-only Revascularization in Elderly Patients with Myocardial Infarction and Multivessel Disease (FIRE) trial showed that among patients >75 years of age with myocardial infarction and multivessel disease, a complete revascularisation was associated with a lower risk of composite death, revealing a clear benefit of PCI for the elderly [14]. Further research about the implications of age-adjusted treatment is needed to investigate the prognostic effect of different therapies, as well as the prognostic value of complete coronary revascularization in patients with HFmrEF.

## 5. Limitations

The main limitations are the retrospective and single-centre study design. Although multivariable risk prediction models were adjusted for potential confounders, individual decision-making (e.g., presence and severity of facility, presumed wishes of the patients) may further affect outcomes;. Additionally, prescription rates of ARNI, MRA, and SGLT2 inhibitors were relatively low, related to the poor evidence in HFmrEF patients. Furthermore, event rates observed in some secondary endpoints were low, potentially reducing the statistical power of the study. Related to the retrospective study design, the risk of recurrent cardiac and HF-related rehospitalization was assessed at our institution only and no information regarding the causes of death beyond index hospitalization were available.

## 6. Conclusions

This study investigated the distribution of HF aetiologies stratified by age and the prognostic impact of age in patients hospitalized with HFmrEF. ICM was the most common cause of HF in patients >40 years of age, whereas NICM was the most common cause in younger patients. Furthermore, higher age was associated with a higher risk of all-cause mortality and HF-related rehospitalization, whereas the adverse effect of age was still observed after adjustment for patients’ comorbidities. Therefore, age-tailored management of HFmrEF is fundamental when considering outcomes in patients and should be further investigated.

## Figures and Tables

**Table 1:** Baseline characteristics.

	Age ≤40 (*n* = 62)	Age >40–≤60 (*n* = 331)	Age >60–≤80 (*n* = 1037)	Age >80 (*n* = 754)	*p* Value across Groups	*p* Value >80 vs. ≤40	*p* Value >80 vs. >40–≤60	*p* Value >80 vs. >60–≤80	*p* Value >60–≤80 vs. ≤40	*p* Value >60–≤80 vs. >40–≤60	*p* Value >40–≤60 vs. ≤40
**Age**, median (IQR)	34 (30–37)	54 (49–57)	72 (67–77)	85 (82–88)	**0.001**	**0.001**	**0.001**	**0.001**	**0.001**	**0.001**	**0.001**
**Male sex**, n (%)	43 (69.4)	256 (77.3)	691 (66.6)	420 (55.7)	**0.001**	0.037 *	**0.001**	**0.001**	0.659	**0.001**	0.176
**Body mass index**, kg/m^2^, median (IQR)	28 (24–34)	29 (25–33)	27 (24–31)	26 (23–29)	**0.001**	**0.001**	**0.001**	**0.001**	0.359	**0.001**	0.665
**SBP**, mmHg, median (IQR)	131 (120–151)	143 (124–163)	143 (125–161)	143 (125–165)	0.192	-	-	-	-	-	-
**DBP**, mmHg, median (IQR)	78 (68–89)	86 (74–99)	80 (70–90)	76 (66–87)	**0.001**	0.257	**0.001**	**0.001**	0.926	**0.001**	0.018 *
**Heart rate**, bpm, median (IQR)	85 (74–101)	84 (73–98)	80 (69–95)	78 (66–93)	**0.001**	**0.006**	**0.001**	**0.008**	0.069	0.021 *	0.512
**Medical history**, n (%)											
Coronary artery disease	4 (6.5)	90 (27.2)	448 (43.2)	354 (46.9)	**0.001**	**0.001**	**0.001**	0.115	**0.001**	**0.001**	**0.001**
Prior myocardial infarction	4 (6.5)	64 (19.3)	259 (25.0)	194 (25.7)	**0.001**	**0.001**	0.023 *	0.717	**0.001**	0.035 *	0.014 *
Prior PCI	4 (6.5)	68 (20.5)	312 (30.1)	228 (30.2)	**0.001**	**0.001**	**0.001**	0.945	**0.001**	**0.001**	**0.008**
Prior CABG	0 (0.0)	14 (4.2)	110 (10.6)	90 (11.9)	**0.001**	**0.004**	**0.001**	0.378	**0.007**	**0.001**	0.099
Prior valvular surgery	5 (8.1)	17 (5.1)	51 (4.9)	23 (3.1)	0.096	-	-	-	-	-	-
Congestive heart failure	11 (17.7)	88 (26.6)	339 (32.7)	303 (40.2)	**0.001**	**0.001**	**0.001**	**0.001**	0.014 *	0.037 *	0.141
Decompensated heart failure <12 months	2 (3.2)	27 (8.2)	118 (11.4)	91 (12.1)	0.054	-	-	-	-	-	-
Prior ICD	1 (1.6)	7 (2.1)	25 (2.4)	9 (1.2)	0.317	-	-	-	-	-	-
Prior sICD	1 (1.6)	3 (0.9)	5 (0.5)	0 (0.0)	0.060	-	-	-	-	-	-
Prior CRT-D	0 (0.0)	3 (0.9)	17 (1.6)	12 (1.6)	0.585	-	-	-	-	-	-
Prior pacemaker	1 (1.6)	7 (2.1)	64 (6.2)	128 (17.0)	**0.001**	**0.001**	**0.001**	**0.001**	0.139	**0.004**	0.797
Chronic kidney disease	5 (8.1)	40 (12.1)	286 (27.6)	348 (46.2)	**0.001**	**0.001**	**0.001**	**0.001**	**0.001**	**0.001**	0.362
Peripheral artery disease	0 (0.0)	23 (6.9)	143 (13.8)	87 (11.5)	**0.001**	**0.005**	0.021 *	0.160	**0.002**	**0.001**	0.032
Stroke	3 (4.8)	20 (6.0)	165 (15.9)	143 (19.0)	**0.001**	**0.005**	**0.001**	0.091	0.019 *	0.001	0.711
Liver cirrhosis	0 (0.0)	4 (1.2)	30 (2.9)	12 (1.7)	0.106	-	-	-	-	-	-
Malignancy	10 (16.1)	26 (7.9)	186 (17.9)	113 (15.0)	**0.001**	**0.001**	0.098	0.718	**0.001**	0.038 *	0.013 *
COPD	1 (1.6)	19 (5.7)	153 (14.8)	90 (11.9)	**0.001**	**0.002**	0.086	**0.004**	**0.001**	0.175	**0.001**
**Cardiovascular risk factors**, n (%)											
Arterial hypertension	13 (21.0)	202 (61.0)	832 (80.2)	655 (86.9)	**0.001**	**0.001**	**0.001**	**0.001**	**0.001**	**0.001**	**0.001**
Diabetes mellitus	6 (9.7)	89 (26.9)	413 (39.8)	291 (38.6)	**0.001**	**0.001**	**0.001**	0.598	**0.001**	**0.001**	**0.004**
Hyperlipidemia	3 (4.8)	91 (27.5)	342 (33.0)	226 (30.0)	**0.001**	**0.001**	0.408	0.177	0.001	0.062	**0.001**
Smoking											
Current	23 (37.1)	141 (42.6)	212 (20.4)	30 (4.0)	**0.001**	**0.001**	**0.001**	0.001	**0.002**	**0.001**	0.420
Former	3 (4.8)	50 (15.1)	220 (21.2)	117 (15.5)	**0.001**	0.022 *	0.863	**0.002**	**0.002**	0.015 *	0.030 *
Family history	11 (17.7)	58 (17.5)	102 (9.8)	30 (4.0)	**0.001**	**0.001**	**0.001**	**0.001**	0.046 *	**0.001**	0.967
**Entry criteria, n (%)**					**0.001**	**0.001**	**0.001**	**0.001**	**0.001**	**0.001**	**0.001**
ACS	14 (22.6)	136 (41.1)	258 (24.9)	138 (18.3)							
Rhythm disorders	12 (19.4)	36 (10.9)	243 (23.4)	205 (27.2)							
ADHF	2 (3.2)	19 (5.7)	157 (15.1)	180 (23.9)							
Pulmonary embolism	0 (0.0)	5 (1.5)	14 (1.4)	10 (1.3)							
Valve disease	3 (4.8)	13 (3.9)	81 (7.8)	77 (10.2)							
Elective procedure	0 (0.0)	4 (1.2)	19 (1.8)	8 (1.1)							
Cardiomyopathy	16 (25.8)	27 (8.2)	42 (4.1)	13 (1.7)							
Chronic coronary syndrome	1 (1.6)	47 (14.2)	144 (13.9)	87 (11.5)							
Others	14 (22.6)	44 (13.3)	79 (7.6)	36 (4.8)							
**Comorbidities at index hospitalization**, n (%)											
Acute coronary syndrome											
Unstable angina	0 (0.0)	19 (5.7)	57 (5.5)	23 (3.1)	**0.018**	0.163	0.034 *	0.013 *	0.058	0.866	0.053
STEMI	6 (9.7)	59 (17.8)	89 (8.6)	22 (2.9)	**0.001**	**0.005**	**0.001**	**0.001**	0.766	**0.001**	0.113
NSTEMI	3 (4.8)	47 (14.2)	117 (11.3)	107 (14.2)	0.056	-	-	-	-	-	-
Acute decompensated heart failure	3 (4.8)	27 (8.2)	211 (20.3)	243 (32.2)	**0.001**	**0.001**	**0.001**	**0.001**	**0.003**	**0.001**	0.366
Cardiogenic shock	3 (4.8)	6 (1.8)	31 (3.0)	13 (1.7)	0.171	-	-	-	-	-	-
Atrial fibrillation	4 (6.5)	57 (17.2)	429 (41.4)	426 (56.5)	**0.001**	**0.001**	**0.001**	**0.001**	**0.001**	**0.001**	0.032 *
Cardiopulmonary resuscitation	2 (3.2)	12 (3.6)	29 (2.8)	10 (1.3)	0.085	-	-	-	-	-	-
Out-of-hospital	1 (1.6)	7 (2.1)	11 (1.1)	3 (0.4)	0.068	-	-	-	-	-	-
In-hospital	1 (1.6)	5 (1.5)	18 (1.7)	7 (0.9)	0.557	-	-	-	-	-	-
Stroke	6 (9.7)	34 (10.3)	139 (13.4)	119 (15.8)	0.072	-	-	-	-	-	-
**Medication at index admission**, n (%)											
ACE inhibitor	6 (9.7)	102 (30.8)	389 (37.5)	278 (36.9)	**0.001**	**0.001**	0.054	0.781	**0.001**	0.027 *	**0.001**
ARB	4 (6.5)	37 (11.2)	252 (24.3)	196 (26.0)	**0.001**	**0.001**	**0.001**	0.414	**0.001**	**0.001**	0.264
Beta blocker	15 (24.2)	127 (38.4)	600 (57.9)	492 (65.3)	**0.001**	**0.001**	**0.001**	**0.002**	**0.001**	**0.001**	0.033 *
MRA	5 (8.1)	30 (9.1)	98 (9.5)	73 (9.7)	0.970	-	-	-	-	-	-
ARNI	1 (1.6)	4 (1.2)	9 (0.9)	5 (0.7)	0.750	-	-	-	-	-	-
SGLT2 inhibitor	0 (0.0)	10 (3.0)	29 (2.8)	6 (0.8)	**0.009**	0.481	**0.005**	**0.003**	0.182	0.831	0.166
Loop diuretics	3 (4.8)	53 (16.0)	393 (37.9)	372 (49.3)	**0.001**	**0.001**	**0.001**	**0.001**	**0.001**	**0.001**	0.021 *
Statin	4 (6.5)	93 (28.1)	513 (49.5)	375 (49.1)	**0.001**	**0.001**	**0.001**	0.912	**0.001**	**0.001**	**0.001**
ASA	5 (8.1)	93 (28.1)	386 (37.2)	251 (33.3)	**0.001**	**0.001**	0.091	0.086	**0.001**	**0.002**	**0.001**
P2Y12 inhibitor	2 (3.2)	29 (8.8)	123 (11.9)	57 (7.6)	**0.005**	0.205	0.500	**0.003**	0.037 *	0.118	0.138
DOAC	4 (6.5)	21 (6.3)	255 (24.6)	240 (31.8)	**0.001**	**0.001**	**0.001**	**0.001**	**0.001**	**0.001**	0.975
Vitamin K antagonist	3 (4.8)	17 (5.1)	86 (8.3)	79 (10.5)	**0.021**	0.156	**0.004**	0.115	0.333	0.058	0.922

ACE, angiotensin-converting-enzyme; ACS, acute coronary syndrome; ADHF, acute decompensated heart failure; ARB, angiotensin receptor blocker; ARNI, angiotensin receptor neprilysin inhibitor; ASA, acetylsalicylic acid; BMI, body mass index; CABG, coronary artery bypass grafting; COPD, chronic obstructive pulmonary disease; CRT-D, cardiac resynchronization therapy with defibrillator; DBP, diastolic blood pressure; DOAC, directly acting oral anticoagulant; IQR, interquartile range; MRA, mineralocorticoid receptor antagonist; (N)STEMI, non-ST-segment elevation myocardial infarction; SBP, systolic blood pressure; SGLT2, sodium glucose linked transporter 2; (s-)ICD, (subcutaneous) implantable cardioverter defibrillator. Level of significance *p* ≤ 0.05. Bold type indicates statistical significance. * According to the Bonferroni method, the α-significance level for the pairwise comparisons was adjusted to *p* ≤ 0.05/6 = 0.008.

**Table 2:** Heart-failure related and procedural data.

	Age ≤40 (*n* = 62)	Age >40–≤60 (*n* = 331)	Age >60–≤80 (*n* = 1037)	Age >80 (*n* = 754)	*p* Value across Groups	*p* Value >80 vs. ≤40	*p* Value >80 vs. >40–≤60	*p* Value >80 vs. >60–≤80	*p* Value >60–≤80 vs. ≤40	*p* Value >60–≤80 vs. >40–≤60	*p* Value >40–≤60 vs. ≤40
**Heart failure etiology**, n (%)											
Ischemic cardiomyopathy	11 (17.7)	184 (55.6)	625 (60.2)	438 (58.2)	**0.001**	**0.001**	**0.001**	**0.003**	**0.001**	0.045 *	**0.001**
Non-ischemic cardiomyopathy	16 (25.8)	37 (11.2)	68 (6.6)	28 (3.7)							
Hypertensive cardiomyopathy	4 (6.5)	31 (9.4)	81 (7.8)	62 (8.2)							
Congenital heart disease	0 (0.0)	2 (0.6)	2 (0.2)	0 (0.0)							
Valvular heart disease	6 (9.7)	9 (2.7)	39 (3.8)	42 (5.6)							
Tachycardia-associated	3 (4.8)	18 (5.4)	53 (5.1)	54 (7.2)							
Tachymyopathy	3 (4.8)	10 (3.0)	15 (1.4)	10 (1.3)							
Pacemaker-induced cardiomyopathy	0 (0.0)	1 (0.3)	5 (0.5)	13 (1.7)							
Unknown	22 (35.5)	49 (14.8)	164 (15.8)	117 (15.5)							
**NYHA functional class**, n (%)											
I/II	60 (96.8)	282 (85.2)	753 (72.6)	490 (65.0)	**0.001**	**0.001**	**0.001**	**0.001**	**0.001**	**0.001**	**0.005**
III	2 (3.2)	37 (11.2)	203 (19.6)	168 (22.3)							
IV	0 (0.0)	12 (3.6)	81 (7.8)	96 (12.7)							
**Echocardiographic data**											
LVEF, %, median (IQR)	46 (45–48)	45 (45–47)	45 (45–47)	45 (45–46)	**0.009**	**0.007**	0.041 *	0.935	0.009 *	0.035 *	0.112
IVSd, mm, median (IQR)	10 (9–12)	11 (10–13)	12 (11–13)	12 (11–13)	**0.001**	**0.001**	**0.002**	0.031 *	**0.001**	0.081	**0.002**
LVEDD, mm, median (IQR)	50 (45–56)	50 (45–55)	49 (44–54)	48 (43–52)	**0.001**	**0.003**	**0.001**	**0.001**	0.142	0.197	0.415
TAPSE, mm, median (IQR)	21 (18–23)	21 (18–23)	20 (18–23)	19 (16–22)	**0.001**	**0.001**	**0.001**	**0.001**	0.039 *	0.035 *	0.297
LA diameter, mm, median (IQR)	36 (33–39)	39 (34–42)	41 (37–47)	45 (39–50)	**0.001**	**0.001**	**0.001**	**0.001**	**0.001**	**0.001**	0.043 *
LA area, cm^2^, median (IQR)	17 (15–20)	18 (15–22)	21 (17–26)	23 (19–27)	**0.001**	**0.001**	**0.001**	**0.001**	**0.004**	**0.001**	0.667
LAVI, mL/m^2^, median (IQR)	24 (19–30)	31 (24–40)	37 (28–49)	45 (36–56)	**0.001**	**0.001**	**0.001**	**0.001**	**0.001**	**0.001**	0.023 *
BSA, cm^2^, median (IQR)	202 (171–224)	198 (181–217)	189 (172–205)	181 (162–195)	**0.001**	**0.001**	**0.001**	**0.001**	0.043 *	**0.001**	0.867
E/A, median (IQR)	1.2 (0.9–1.3)	0.9 (0.7–1.2)	0.8 (0.6–1.1)	0.8 (0.6–1.2)	**0.001**	**0.001**	**0.001**	0.415	**0.001**	**0.001**	**0.005**
E/E‘, median (IQR)	7.0 (4.0–10.0)	8.5 (5.5–11.0)	9.0 (6.5–13)	11.5 (7.3–16.0)	**0.001**	**0.001**	**0.001**	**0.001**	0.011 *	0.009 *	0.191
Diastolic dysfunction, n (%)	18 (29.0)	210 (63.4)	767 (74.0)	579 (76.8)	**0.001**	**0.001**	**0.001**	0.172	**0.001**	**0.001**	**0.001**
Moderate–severe AS, n (%)	1 (1.6)	0 (0.0)	71 (6.8)	142 (18.8)	**0.001**	**0.001**	**0.001**	**0.001**	0.106	**0.001**	0.021 *
Moderate–severe AR, n (%)	2 (3.2)	6 (1.8)	33 (3.2)	43 (5.7)	**0.007**	0.411	**0.004**	0.009 *	0.985	0.192	0.470
Moderate–severe MR, n (%)	1 (1.6)	13 (3.9)	103 (9.9)	145 (19.2)	**0.001**	**0.001**	**0.001**	**0.001**	0.030 *	**0.001**	0.367
Moderate–severe TR, n (%)	1 (1.6)	9 (2.7)	126 (12.2)	208 (27.6)	**0.001**	**0.001**	**0.001**	**0.001**	0.012 *	**0.001**	0.612
VCI, median (IQR)	16 (15–17)	18 (13–23)	19 (14–25)	22 (17–26)	**0.020**	**0.003**	0.009 *	0.023 *	0.093	0.273	0.338
Aortic root, mm, median (IQR)	31 (28–34)	33 (30–36)	33 (30–36)	33 (29–36)	**0.003**	0.026 *	0.518	0.098	**0.004**	0.531	0.012 *
**Coronary angiography,** n (%)	18 (29.0)	185 (55.9)	477 (46.0)	220 (29.2)	**0.001**	0.981	**0.001**	**0.001**	0.009 *	**0.002**	**0.001**
No evidence of CAD	9 (50.0)	43 (23.2)	89 (18.7)	34 (15.5)	**0.001**	**0.001**	0.045 *	0.775	**0.001**	0.097	0.009 *
1-vessel disease	6 (33.3)	41 (22.2)	81 (17.0)	38 (17.3)							
2-vessel disease	3 (16.7)	39 (21.1)	102 (21.4)	48 (21.8)							
3-vessel disease	0 (0.0)	62 (33.5)	205 (43.0)	100 (45.5)							
CABG	0 (0.0)	8 (4.3)	43 (9.0)	22 (10.0)	0.081	0.685	0.308	**0.005**	0.631	0.351	0.010 *
Chronic total occlusion	1 (5.6)	21 (11.4)	67 (14.0)	67 (14.0)	0.465	0.476	0.888	0.253	0.304	0.359	0.450
PCI, n (%)	9 (50.0)	109 (58.9)	251 (52.6)	112 (50.9)	0.390	0.941	0.107	0.674	0.827	0.144	0.464
Sent to CABG, n (%)	0 (0.0)	12 (6.5)	32 (6.7)	7 (3.2)	0.185	0.442	0.117	0.060	0.256	0.918	0.265
Baseline laboratory values, median (IQR)											
Potassium, mmol/L	3.9 (3.7–4.1)	3.9 (3.7–4.2)	3.9 (3.6–4.2)	3.9 (3.5–4.2)	0.170	-	-	-	-	-	-
Sodium, mmol/L	138 (137–140)	139 (137–141)	139 (137–141)	139 (137–141)	0.167	-	-	-	-	-	-
Creatinine, mg/dL	0.90 (0.77–1.03)	0.96 (0.82–1.14)	1.07 (0.85–1.42)	1.18 (0.95–1.63)	**0.001**	**0.001**	**0.001**	**0.001**	**0.001**	**0.001**	0.128
eGFR, mL/min/1.73 m^2^	95 (82–107)	85 (68–97)	67 (47–86)	54 (37–71)	**0.001**	**0.001**	**0.001**	**0.001**	**0.001**	**0.001**	**0.001**
Hemoglobin, g/dL	13.6 (11.7–15.1)	13.9 (12.0–14.9)	12.3 (10.3–14.0)	11.9 (10.1–13.3)	**0.001**	**0.001**	**0.001**	**0.001**	**0.001**	**0.001**	0.879
WBC count, × 10^9^/L	8.52 (6.72–11.66)	8.99 (6.97–10.52)	8.13 (6.40–10.03)	7.93 (6.32–10.04)	**0.003**	0.159	**0.001**	0.434	0.252	**0.002**	0.785
Platelet count, × 10^9^/L	262 (183–331)	241 (203–292)	224 (175–282)	219 (175–276)	**0.001**	0.013 *	**0.001**	0.299	0.031 *	**0.001**	0.432
HbA1c, %	5.3 (5.0–5.8)	5.8 (5.4–6.7)	5.9 (5.5–6.8)	5.9 (5.6–6.8)	**0.001**	**0.001**	0.017 *	0.730	**0.001**	0.056 *	**0.001**
LDL cholesterol, mg/dL	107 (82–131)	121 (93–148)	97 (75–126)	87 (68–113)	**0.001**	**0.006**	**0.001**	**0.001**	0.153	**0.001**	0.184
HDL cholesterol, mg/dL	41 (35–49)	39 (31–47)	42 (34–52)	44 (36–54)	**0.001**	0.151	**0.001**	0.009 *	0.629	**0.003**	0.365
C-reactive protein, mg/L	14 (3–34)	10 (3–25)	12 (4–46)	17 (5–50)	**0.001**	0.260	**0.001**	0.019 *	0.735	**0.001**	0.151
NT-proBNP, pg/mL	383 (77–2369)	691 (204–2196)	2620 (1066–5881)	4124 (2040–9169)	**0.001**	**0.001**	**0.001**	**0.001**	**0.001**	**0.001**	0.193
Cardiac troponin I, µg/L	0.02 (0.02–0.25)	0.03 (0.02–0.45)	0.03 (0.02–0.23)	0.03 (0.02–0.12)	0.365	-	-	-	-	-	-
**Medication at discharge**, n (%)											
ACE inhibitor	28 (45.9)	189 (57.4)	513 (51.0)	328 (46.0)	**0.005**	0.988	**0.001**	0.041 *	0.440	0.042 *	0.096
ARB	5 (8.2)	50 (15.2)	246 (24.5)	198 (27.8)	**0.001**	**0.001**	**0.001**	0.122	**0.004**	**0.001**	0.149
Beta blocker	40 (65.6)	246 (74.8)	797 (79.2)	552 (77.4)	**0.044**	0.036 *	0.348	0.370	0.012 *	0.090	0.136
MRA	9 (14.8)	42 (12.8)	149 (14.8)	96 (13.5)	0.763	0.778	0.757	0.431	0.990	0.358	0.672
ARNI	3 (4.9)	5 (1.5)	11 (1.1)	6 (0.8)	**0.039**	**0.004**	0.319	0.603	0.011 *	0.537	0.085
SGLT2 inhibitor	1 (1.6)	19 (5.8)	50 (5.0)	14 (2.0)	**0.003**	0.860	**0.001**	**0.001**	0.236	0.567	0.179
Loop diuretics	9 (14.8)	80 (24.3)	484 (48.1)	445 (62.4)	**0.001**	**0.001**	**0.001**	**0.001**	**0.001**	**0.001**	0.102
Statin	12 (19.7)	215 (65.3)	727 (72.3)	488 (68.4)	**0.001**	**0.001**	0.322	0.086	**0.001**	0.017 *	**0.001**
Digitalis	0 (0.0)	6 (1.8)	48 (4.8)	49 (6.9)	**0.001**	0.034 *	**0.001**	0.063	0.081	0.018 *	0.288
Amiodarone	1 (1.6)	6 (1.8)	27 (2.7)	24 (3.4)	0.500	0.464	0.166	0.411	0.620	0.383	0.921
ASA	16 (26.2)	197 (59.9)	533 (53.0)	317 (44.5)	**0.001**	**0.006**	**0.001**	**0.001**	**0.001**	0.029 *	**0.001**
P2Y12 inhibitor	10 (16.4)	131 (39.8)	355 (35.3)	172 (24.1)	**0.001**	0.172	**0.001**	**0.001**	**0.003**	0.138	**0.001**
DOAC	8 (13.1)	37 (11.2)	336 (33.4)	309 (43.3)	**0.001**	**0.001**	**0.001**	**0.001**	**0.001**	**0.001**	0.675
Vitamin k antagonist	3 (4.9)	17 (5.2)	76 (7.6)	54 (7.6)	0.414	-	-	-	-	-	-

ACE, angiotensin-converting enzyme; AR, aortic regurgitation; ARB, angiotensin receptor blocker; ARNI, angiotensin receptor neprilysin inhibitor; AS, aortic stenosis; ASA, acetylsalicylic acid; BSA, body surface area; CABG, coronary artery bypass grafting; CAD, coronary artery disease; DOAC, directly acting oral anticoagulant; eGFR, estimated glomerular filtration rate; HbA1c, glycated haemoglobin; HDL, high-density lipoprotein; IQR, interquartile range; IVSd, Interventricular septal end diastole; LA, left atrial; LAVI, left atrial volume index; LDL, low-density lipoprotein; LVEDD, Left ventricular end-diastolic diameter; LVEF, left ventricular ejection fraction; MR, mitral regurgitation; MRA, mineralocorticoid receptor antagonist; NT-proBNP, amino terminal pro-B-type natriuretic peptide; NYHA, New York Heart Association; PCI, percutaneous coronary intervention; SGLT2, sodium glucose linked transporter 2; TAPSE, tricuspid annular plane systolic excursion; TR¸ tricuspid regurgitation; VCI, vena cava inferior; WBC, white blood cells. Level of significance *p* ≤ 0.05. Bold type indicates statistical significance. * According to the Bonferroni method, the α-significance level for the pairwise comparisons was adjusted to *p* ≤ 0.05/6 = 0.008.

**Table 3:** Follow-up data, primary and secondary endpoints.

	Age ≤40 (*n* = 62)	Age >40–≤60 (*n* = 331)	Age >60–≤80 (*n* = 1037)	Age >80 (*n* = 754)	*p* Value across Groups	*p* Value >80 vs. ≤40	*p* Value >80 vs. >40–≤60	*p* Value >80 vs. >60–≤80	*p* Value >60–≤80 vs. ≤40	*p* Value >60–≤80 vs. >40–≤60	*p* Value >40–≤60 vs. ≤40
**Primary endpoint**, n (%)											
All-cause mortality, at 30 months	7 (11.3)	35 (10.6)	279 (26.9)	362 (48.0)	**0.001**	**0.001**	**0.001**	**0.001**	**0.006**	**0.001**	0.867
**Secondary endpoints**, n (%)											
All-cause mortality, in-hospital	1 (1.6)	2 (0.6)	31 (3.0)	41 (5.4)	**0.031**	0.190	**0.001**	0.009 *	0.531	0.014 *	0.402
All-cause mortality, at 12 months	5 (8.1)	19 (5.7)	190 (18.3)	252 (33.4)	**0.001**	**0.001**	**0.001**	**0.001**	0.040 *	**0.001**	0.483
Heart failure related rehospitalization, at 30 months	3 (4.9)	14 (4.3)	147 (14.6)	115 (16.1)	**0.001**	0.019 *	**0.001**	0.389	0.034 *	**0.001**	0.816
Cardiac rehospitalization, at 30 months	7 (11.5)	52 (15.8)	255 (25.3)	148 (20.8)	**0.001**	0.082	0.059	0.027 *	0.015 *	**0.001**	0.386
Coronary revascularization, at 30 months	2 (3.3)	33 (10.0)	80 (8.0)	27 (3.8)	**0.001**	0.841	**0.001**	**0.001**	0.183	0.240	0.090
Acute myocardial infarction, at 30 months	0 (0.0)	6 (1.8)	41 (4.1)	17 (2.4)	**0.017**	0.223	0.567	0.056	0.108	0.054	0.288
Stroke, at 30 months	2 (3.3)	10 (3.0)	25 (2.5)	20 (2.8)	0.930	-	-	-	-	-	-
MACCE, at 30 months	10 (16.1)	73 (22.1)	369 (35.6)	389 (51.6)	**0.001**	**0.001**	**0.001**	**0.001**	**0.002**	**0.001**	0.294
**Follow-up data**, median (IQR)											
Hospitalization time, days	7 (4–10)	6 (4–11)	9 (6–16)	10 (6–17)	**0.001**	**0.001**	**0.001**	0.022 *	**0.001**	**0.001**	0.914
ICU time, days	0 (0–1)	0 (0–1)	0 (0–1)	0 (0–0)	**0.001**	0.068	**0.001**	**0.001**	0.880	0.512	0.889
Follow-up time, days	1289 (647–2063)	1376 (698–2010)	998 (432–1756)	638 (196–1221)	**0.001**	**0.001**	**0.001**	**0.001**	0.029 *	**0.001**	0.766

ICU, intensive care unit; MACCE, major adverse cardiac and cerebrovascular events. Level of significance *p* ≤ 0.05. Bold type indicates statistical significance.

**Table 4:** Multivariate Cox regression analyses with regard to all-cause mortality and heart failure-related rehospitalization at 30 months.

	30-Months All-Cause Mortality			Heart Failure-Related Rehospitalization		
	HR	95% CI	*p* Value	HR	95% CI	*p* Value
Sex	1.195	0.997–1.433	0.054	0.854	0.653–1.117	0.250
BMI (per 1 kg/m^2^ increase)	0.952	0.934–0.970	**0.001**	1.021	0.997–1.046	0.084
Prior chronic heart failure	1.223	1.021–1.466	**0.029**	2.087	1.594–2.732	**0.001**
Arterial hypertension	0.882	0.703–1.107	0.277	1.232	0.824–1.842	0.310
Diabetes mellitus	1.254	1.046–1.505	**0.015**	1.318	1.004–1.731	**0.047**
Hyperlipidemia	0.705	0.577–0.861	**0.001**	0.822	0.621–1.090	0.173
Creatinine, (per 1 mg/dL increase)	1.156	1.091–1.226	**0.001**	1.111	1.013–1.219	**0.026**
Malignancy	3.099	2.564–3.746	**0.001**	0.853	0.575–1.265	0.429
Ischemic cardiomyopathy	0.770	0.646–0.918	**0.004**	1.287	0.972–1.703	0.078
Atrial fibrillation	1.183	0.981–1.426	0.078	1.913	1.432–2.557	**0.001**
Acute decompensated heart failure	1.611	1.335–1.942	**0.001**	2.023	1.536–2.664	**0.001**
Cardiogenic shock	2.556	1.598–4.087	**0.001**	2.070	0.963–4.452	0.062
TAPSE <18 mm	1.262	1.047–1.521	**0.014**	1.080	0.813–1.434	0.594
Aortic stenosis	1.416	1.116–1.796	**0.004**	1.682	1.181–2.395	**0.004**
Aortic regurgitation	0.991	0.702–1.398	0.957	1.501	0.920–2.450	0.104
Mitral regurgitation	1.003	0.796–1.264	0.981	1.323	0.937–1.867	0.112
Tricuspid regurgitation	1.431	1.154–1.773	**0.001**	1.130	0.810–1.577	0.471
Age >40–≤60 years	1.060	0.462–2.435	0.890	0.516	0.146–1.824	0.304
Age >60–≤80years	2.211	1.020–4.790	0.044	1.037	0.317–3.394	0.952
Age >80 years	3.874	1.770–8.478	**0.001**	0.833	0.249–2.784	0.766
Age ≤40 years	(reference group)			(reference group)		
Age (per decade increase) *	1.416	1.301–1.541	**0.001**	1.077	0.950–1.221	0.247
Age (per year increase) **	1.037	1.028–1.046	**0.001**	1.008	0.995–1.021	0.223

CI, confidence interval; HR, hazard ratio; BMI, body mass index; TAPSE, tricuspid annular plane systolic excursion; NYHA, New York Heart Association. * Multivariable Cox regression analyses were additionally performed with age per decade (*) and per year (**) increase. Level of significance *p* ≤ 0.05. Bold type indicates statistical significance.

**Table 5:** Hazard ratios for age within pre-specified subgroups after multivariable adjustment.

	30-Months All-Cause Mortality			Heart Failure-Related Rehospitalization		
	HR	95% CI	*p* Value	HR	95% CI	*p* Value
Age > 75	1.288	1.041–1.594	**0.020**	1.430	1.022–2.001	**0.037**
Age ≤ 75	1.086	0.764–1.544	0.646	0.969	0.613–1.533	0.893
Male sex	1.245	0.993–1.561	0.058	1.200	0.848–1.699	0.303
Female sex	1.257	0.931–1.698	0.136	1.500	0.980–2.296	0.062
Ischemic cardiomyopathy	1.228	1.128–1.419	0.107	1.414	1.010–1.980	**0.044**
No ischemic cardiomyopathy	1.250	0.957–1.632	0.102	1.254	0.797–1.974	0.328
NYHA functional class ≤2	1.332	1.055–1.680	**0.016**	1.303	0.894–1.899	0.169
NYHA functional class >2	1.075	0.804–1.439	0.625	1.392	0.947–2.046	0.092
TAPSE ≥18 mm	1.271	1.010–1.600	**0.041**	1.221	0.873–1.707	0.243
TAPSE <18 mm	1.271	0.940–1.717	0.119	1.538	0.976–2.423	0.064

CI, confidence interval; GFR, glomerular filtration rate; HR, hazard ratio; NYHA, New York Heart Association; TAPSE, tricuspid annular plane systolic excursion. Level of significance *p* ≤ 0.05. Bold type indicates statistical significance. Multivariable Cox regression models were adjusted for age, sex, body mass index, coronary artery disease, prior acute myocardial infarction, chronic kidney disease, malignancy, arterial hypertension, atrial fibrillation, acute myocardial infarction, ischemic cardiomyopathy, NYHA functional class, TAPSE, and the presence of diabetes mellitus.

## Data Availability

The dataset used and/or analysed during the current study is available from the corresponding author upon reasonable request.
